# Effect of e-service quality on customer engagement behavior in community e-commerce

**DOI:** 10.3389/fpsyg.2022.965998

**Published:** 2022-09-08

**Authors:** Wenfang Fan, Bingjia Shao, Xiaohua Dong

**Affiliations:** ^1^School of Economics and Business Administration, Chongqing University, Chongqing, China; ^2^School of Economics and Business Administration, Chongqing Key Laboratory of Logistics, Chongqing University, Chongqing, China

**Keywords:** community e-commerce, e-service quality, customer engagement behavior, customer trust, perceived risk

## Abstract

Customer engagement behavior is a critical success factor for community e-commerce. While many community e-commerce websites are currently improving service quality to enhance customer engagement behavior, little is known about how such e-services affect customer engagement behavior. Building upon the *stimulus-organism-response* (SOR) model, this study developed a research model to explain how e-service quality of community e-commerce platform affects customer engagement behavior through customer trust and perceived risk. The research model was empirically evaluated by surveying 326 customers who have shopped through the community e-commerce platforms. The results indicate that e-service quality (system design, intelligent fulfillment, security assurance, and interactive service) positively affects customer engagement behavior. Besides, customer trust and perceived risk play a mediating role between e-service quality and customer engagement behavior. This study offers recommendations to managers on how to build an attractive community e-commerce platform to stimulate customer engagement behavior.

## Introduction

The emergence of e-commerce platform has changed the way of traditional retail trading, fundamentally breaking the limitation of time and space, and providing enterprises and customers with a convenient interactive platform. It is more convenient for customers to realize their purchasing and social needs on the e-commerce platform, and customer engagement behavior has become a critical determinant for the success of e-commerce platforms (Roy et al., 2018a). In highly competitive online shopping environments, providing high e-service quality to promote customer engagement behavior is a major challenge for online enterprises (Vo et al., 2020). Briefly defined, the core of customer engagement is that customers are highly participated in the interactive process, which contributes to the development of customer-based outcomes, such as customer loyalty, and helps to improve the competitive advantage of enterprises (Van Doorn et al., 2010). It is thus important for e-commerce platforms to understand how to stimulate customer engagement behavior (Roy et al., 2018a).

Studies in the e-commerce and marketing domain indicate that the characteristics of e-commerce websites such as interactivity and personalization can positively affect customer engagement behavior (Benlian, 2015; An et al., 2020; Henkens et al., 2021). Inspired by the service characteristics of successful e-commerce, many community companies have begun to improve the service quality of the platform and integrate virtual community functions into e-commerce websites to increase their attractiveness to consumers (Goraya et al., 2021). *Community e-commerce* involves an interactive and sharing e-commerce model, which drives the completion of customers’ purchase transactions through social interaction, information sharing, and content generation among members in a virtual community (Wang et al., 2021). Prominent characteristics of community commerce e-services are personalization and communization (Luo et al., 2020).

Through e-services, customers can form the perception of e-service quality, thus leading to a change in customer behavior (Liu et al., 2021; Cheng et al., 2022). For instance, customers can join personalized virtual communities through the interactive interface, including interest circles and group buying, which greatly improves customers’ purchase behavior (Chen et al., 2015). Furthermore, personalized recommendations and high participation in e-service promote customers’ trust, thereby also influencing customer engagement behavior (Chiang et al., 2020). As such e-services can help customers make behavioral decisions, and they will attach importance to the e-service quality of community e-commerce platform. On the other hand, with the improvement of e-service quality, enterprises can improve customer recognition, a favorable corporate reputation, observed corporate growth and customer loyalty, thus promoting enterprises to achieve sustainable social practice (Fernando et al., 2022). Importantly, promoting customer engagement behavior is an important strategy for enterprises to improve customer loyalty, as well as an important way to promote value co-creation and knowledge sharing between customers and enterprises (Anand et al., 2020). Therefore, it is crucial for the community e-commerce platform to improve the e-service quality and stimulate customer engagement behavior.

Yet, although some studies provided initial evidence that a positive causal relationship may, exist between the e-service quality of e-commerce platform and customer behavior (Elsharnouby and Mahrous, 2015; Al-dweeri et al., 2017), other studies on virtual communities also found that the cognitive internal states and attitudes of customers may significantly affect customers’ engagement (Brengman and Karimov, 2012; Vohra and Bhardwaj, 2019). However, the studies on customer engagement behavior of e-commerce platforms fail to consider the antecedent effect of e-service quality in detail. The present literature does not explain why e-service quality may influence customer engagement behavior, especially for community e-commerce platforms.

While customers’ perceptions of platforms, such as trust and perceived risks, seem to play a significant role in the formation of customer engagement behavior (Roy et al., 2018a), only few studies have examined the influence of e-service quality on these factors. Luo et al. (2020) have analyzed how e-service quality affects customer trust in online second-hand exchange platform. Mou et al. (2020) have studied how alternative website quality affects customer trust and perceived risk. The results of the studies indicate that e-service quality may have different influences on customer perception factors according to its dimensional characteristics. As only the e-service quality of traditional e-commerce has been investigated, it remains unclear how the e-service quality of community e-commerce platform may affect customer trust and perceived risk and the resulting customer engagement behavior.

To better understand if and how the e-service quality of community e-commerce platform influences customer engagement behavior, this study systematically explored the effects generated by the e-service quality of community e-commerce platform (system design, intelligent fulfillment, security assurance, and interactive service). This study is guided by two research questions: *(RQ1) What impact does the e-service quality of community e-commerce platforms have on customer engagement behavior?* To explain the impact of e-service quality, this study developed a research model that connects the e-service quality of community e-commerce platforms (system design, intelligent fulfillment, security assurance, and interactive service) to customer engagement behavior, through customer trust and perceived risk. In so doing, this study investigated: *(RQ2) How do customer trust and perceived risk mediate the relationship between e-service quality and customer engagement behavior?*

Previous studies have suggested that the *stimulus-organism-response* (SOR) model can be used to understand the effect of websites’ characteristics on customer behaviors (Benlian, 2015; Lin et al., 2017). According to the SOR model, customer perceptions (O) are influenced by the external environment (S), especially e-service quality, which eventually drives their different behaviors (R). Thus, this study used the SOR model as an overarching framework to describe the causal relationship between the e-service quality of community e-commerce platform, customer trust and perceived risk, and customer engagement behavior. This study conducted a survey among customers of the community e-commerce platform, and 326 participants used and reported the results of the questionnaire.

The results of this study provide novel contributions to the research stream on customer engagement behavior and community e-commerce. On the one hand, this research contributes insights to answer the question of customer engagement behavior, which is a crucial factor for the success of a community e-commerce platform and can be strengthened by high e-service quality. Up to now, this question has not been examined although it is crucial (Vo et al., 2020). The developed research model considers the e-service characteristics of community e-commerce platforms and introduces the e-service quality as a determinant of consumer perceptions and behavioral responses. On the other hand, this research provides a research model to study and explore how customer perception factors and their interplay mediate the relationship between e-service quality and customer engagement behavior. So far, early e-service quality studies have mainly focused on studying how e-service quality can affect customer satisfaction. This research model considers community influence, and introduces customer trust and perceived risk as important perceived factors of community e-commerce, and hence provides a step toward studying community e-commerce activities from a more holistic perspective.

## Literature review

### Community e-commerce

Community e-commerce integrates virtual communities with e-commerce (Luo et al., 2020). It promotes communication and interaction among customers of e-commerce platforms through virtual communities, and thus drives business transactions (Goraya et al., 2021). Community e-commerce has changed traditional e-commerce in several ways. On the one hand, a community e-commerce platform provides many personalized community functions, which is an important way to attract customers. Through highly interactive social activities, customers can be more motivated to participate, which also makes community e-commerce closer to customers (Wang et al., 2021). On the other hand, traditional e-commerce mainly relies on large-scale traffic to provide competitiveness for business development, while community e-commerce mainly realizes market segmentation based on virtual communities. The precise positioning of customers can be achieved through community marketing, thus improving customer conversion rate (Chen et al., 2015).

Previous studies on community e-commerce mainly focused on online group buying (one of the community e-commerce) and virtual community (Yen and Chang, 2015; Zhang et al., 2021). However, several recent studies have offered fresh insights into community e-commerce (Vohra and Bhardwaj, 2019; Wang et al., 2021). Some scholars have adopted the perspective of satisfaction to study which factors determine customers’ continuance usage intention in community e-commerce platforms (An et al., 2020). Other researchers have studied the influence of motivation and attitude on customers’ willingness to participate in online group buying (Luo et al., 2020).

However, because community e-commerce shopping entails significant human-computer interaction and interpersonal interaction, it is necessary to consider both the e-service and customer perception thereof together. In the community e-commerce literature, preliminary evidence suggests that the e-services of community e-commerce platforms may affect consumers differently depending on their unique needs (Vohra and Bhardwaj, 2019). However, few studies have examined how the e-service of community e-commerce platform influences customers’ repeat purchase behavior and social interaction. Therefore, this study comprehensively considers the e-service quality and customer perception of community e-commerce and explores the changes in the influence of e-service quality on customer engagement behavior.

### The stimulus-organism-response model

The SOR model emphasizes that certain factors in the environment (stimulus) influence the internal emotional and cognitive states of an individual (organism), and thereby influence the individual’s behavior (response) (Mehrabian and Russell, 1974). According to the SOR model, the perceived state of the organism mediates the relationship between stimuli and customer behavior (Kang et al., 2021). In the e-commerce domain, several studies adopted the SOR model to examine how website characteristics as stimulus (e.g., platform environment characteristics and service quality) affect customer behavior, such as their purchasing behavior (Benlian, 2015; Liu et al., 2021). Similarly, Lin et al. (2017) used the SOR model to study which and how technical features of social commerce websites can affect the repurchase intentions of customers.

Given the different perspectives of these studies, various factors have been suggested to measure customers’ perceived state, such as trust and perceived risk. From the results of these studies, the SOR model not only is well suitable for explaining how a certain website stimulus (such as e-service quality) affects customer perception, and then affects customer engagement behavior. By establishing a causal relationship between stimulus, organism, and response, it also provides a reasonable mechanism to track the effects caused by the e-service quality of community e-commerce platform. The e-services of community e-commerce platforms are designed to stimulate specific cognitive perceptions of customers to increase purchase intention and social interaction (Luo et al., 2020). Numerous intelligent e-service functions, such as community activities and group buying (Yen and Chang, 2015), meet the personalized consumption needs of customers that promote customer interaction and the formation of customer community belonging. All of these factors influence customer purchase intention and social interaction. Following the SOR model, we operationalize “stimulus” as the e-service quality of community e-commerce platforms (system design, intelligent fulfillment, security assurance, and interactive service), “organism” as customer trust and perceived risk, and “response” as customer engagement behavior.

### E-service quality

Service quality has been widely recognized as a prerequisite for customer perception (Luo et al., 2020). E-service quality refers to “the extent to which online websites improve the efficiency and effectiveness of customers’ browsing and consumption, including service links such as distribution and consultation” (Zeithaml et al., 2000). It reflects customers’ overall quality perception of the online transaction process and service results (Gummerus et al., 2004). E-service quality has been extensively studied in customer perception literature, mainly involving the influence of perceived quality on business-customer behavior relationships (Hung et al., 2014). For example, Elsharnouby and Mahrous (2015) argue that the e-service quality affects customers’ attitudes toward websites and their willingness to participate.

E-service quality has different dimensions, suggesting that there are different ways to influence customer perception in a specific environment. Wolfinbarger and Gilly (2003) combined the concept of static quality with the dynamic measurement method and proposed the E-Tail Q model, including website design, performance/reliability, privacy/security, and customer service. In addition, based on the virtual environment, some researchers divide e-service quality into four dimensions and propose an E-S-QUAL model: efficiency, system availability, fulfillment, and privacy (Parasuraman et al., 2005).

Lopes et al. (2019) found that compared with the E-S-QUAL model when measured by the E-Tail Q scale, perceived quality can better explain positive word-of-mouth and repurchase intention. The E-Tail Q model is more suitable to explain customers’ perception of service provider commitment (McMullan, 2005). Since this research is to explore the influence of e-service quality on customers’ repeat purchase behavior and social interaction behavior, this research focuses on revealing customers’ perceptions and evaluations of community e-commerce service providers, and the research scenario is more similar to the E-Tail Q model. Therefore, this research uses the dimension design of e-service quality developed by Wolfinbarger and Gilly (2003) for reference to illustrate the comprehensive evaluation of e-service by customers in all steps of the purchase process on the e-commerce platform.

Referring to the dimension design of the eTailQ model, combined with the e-service characteristics of community e-commerce, e-service quality is divided into four factors. *System design*, which provides the basis for all other dimensions of e-service quality, is composed of functions that provide customers with personalized interface presentations, covering all processes of customer page browsing, information search, and community selection (Lopes et al., 2019). For instance, commodity exchange is conducted through the community function in the platform interface. *Intelligent fulfillment* includes the accurate display and description of commodity information, and the ability of the e-commerce platform to deliver purchased commodities within the promised time, emphasizing the ability to use big data for intelligent analysis (Elsharnouby and Mahrous, 2015), for example, tracking the logistics information of commodities through big data analysis. *Security assurance* refers to the safety degree of an e-commerce platform to protect customers’ consumption experience (Luo et al., 2020), for example, through privacy agreements or anonymous commenting functions. *Interactive service* includes the services that the community e-commerce platform can quickly and effectively respond to customers’ purchase transactions, consultation, and assistance needs (Ahmad et al., 2017), such as responding, helping, and answering customers’ questions.

### Customer trust and perceived risk

To represent the cognitive states (of the organism), this study draws on two factors, customer trust and perceived risk. Literature suggests that customer trust and perceived risk can significantly influence customer behavior (Shao et al., 2019; Andronie et al., 2021; Goraya et al., 2021). Moreover, the website service characteristics of an e-commerce platform may have an impact on these factors (Jiang and Lau, 2021).

Trust appears between two parties who act consciously in altruism (Mayer et al., 1995). In a specific environment, trust can be transferred between multiple trust subjects in the environment, and the trust parties can actively understand the trust objects through contact and interaction, so as to realize trust transfer (Salganik et al., 2006). Research shows that potential customers of e-commerce platforms can transfer their perception of trust in the shopping environment of the platform website to the relevant subjects of the goods they want to purchase, stimulating and improving their purchase and consumption intentions (Jiang and Lau, 2021). In addition, several e-commerce studies have shown that trust can significantly increase consumers’ online purchasing behavior and/or information sharing behavior (Chen and Chang, 2012; Lu et al., 2016; Vinerean et al., 2022). This study adopts the definition of customer trust (McKnight et al., 2002), which focuses on the customer’s trust in the e-commerce platform, and emphasizes that customers believe in the e-commerce platform and hold objective trust expectations.

Perceived risk is an uncertain subjective psychological evaluation produced by customers in the purchase decision process (Taylor, 1974). This uncertainty mainly comes from two reasons, the lack of relevant knowledge and the interference of uncontrollable factors (Sitkin and Pablo, 1992). Some studies have shown that the higher the level of uncertainty, the stronger the perceived risk (Chen and Chang, 2012; Liang et al., 2018). In addition, uncontrollable factors increase with the virtuality of the network environment, and these uncertainties all increase the perceived risks (Shao et al., 2019; Paz and Delgado, 2020). This study focus on the customer’s expectation perception of loss in the process of using the e-commerce platform, including the subjective psychological perception of various possible consequences after consumption.

Although many studies have explored the role of customer trust and perceived risk in the field of e-commerce and e-service (Hansen et al., 2018; Vimalkumar et al., 2021), most of them have not focused on the community e-commerce platform to explore the intrinsic role of trust mechanism and perceived risk. For the development of a community e-commerce platform, the sense of trust and belonging of community members is crucial to their purchasing behavior and social interaction behavior. Improving customers’ sense of trust and belonging and reducing their perceived risks can greatly promote customer engagement behavior. However, the mechanism of risk and trust of community e-commerce platform has not been well studied. Therefore, this study regards customer trust and perceived risk as motivating factors of customer behavior, which are affected by the e-service quality of community e-commerce.

### Customer engagement behavior

Customer engagement behavior is a critical factor for e-commerce platform to realize value co-creation (Mollen and Wilson, 2010). Customer engagement has broadly been conceptualized from different perspectives. On the one hand, customer engagement is conceptualized as the psychological process of building a brand relationship through customers’ cognition and emotion (Bowden, 2009; Henkens et al., 2021). Jaakkola and Matthew (2014) believe that customer engagement refers to the psychological state of customers who participate in the process of value co-creation through interaction. On the other hand, customer engagement is conceptualized as a series of behaviors are driven by motivation factors (Kang et al., 2021). Van Doorn et al. (2010) claimed that customer engagement behavior is a series of behaviors generated by customers in the experience process, not just purchase behavior. These behaviors may bring value to the company and ensure its future sales and performance (Cambra-Fierro et al., 2013).

Customer engagement behavior is usually expressed as the spontaneous behavior of customers, and it is the behavioral manifestation of customers participating in the process of value co-creation (Van Doorn et al., 2010). In the process of mutual cooperation and interaction between customers and enterprises, and between customers and customers, value is created together, and psychological and behavioral customer engagement is formed (Centobelli et al., 2020). Therefore, studies using this conceptualization typically measure customer engagement behavior through different behavioral indicators. Some scholars mainly measure customer engagement behaviors through a large number of social interaction behaviors, such as word-of-mouth activities, recommendations, helping other customers, and sharing and forwarding comments (Van Doorn et al., 2010; Chiang et al., 2020). When firms provide more convenient and fair services, positive word-of-mouth behaviors and helping behaviors of customers will appear (Roy et al., 2018a,b). Cambra-Fierro et al. (2013) claimed that customer engagement is a series of transactional (repurchase and loyalty) and non-transactional behaviors (commitment, word-of-mouth, and customer-to-customer recommendations). Given its broad scope, driven by previous literature, this study emphasizes that customer engagement behavior mainly refers to customer repeat purchase behavior in the process of consumer experience and value co-creation, and customer social interaction behavior, such as comment interaction, and word-of-mouth recommendation, and helping others.

## Research hypotheses

**FIGURE 1:**
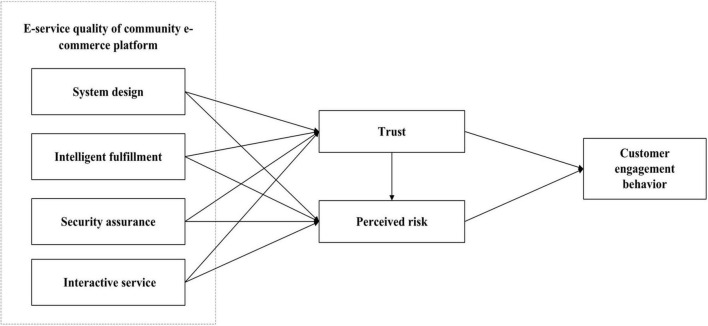
Research model.

Considering that customer engagement behavior in community e-commerce relies on e-services to participate in the interaction process, not just customer perception. Therefore, our study combines a stimulus-organism-response model to test critical antecedents that impact customer engagement behavior on community e-commerce platform. Specifically, the e-service quality (system design, intelligent fulfillment, security assurance, and interactive service) of community e-commerce platform acts as stimulus to influence customer trust and perceived risk (O), which subsequently affects customer engagement behavior (R). Additionally, since trust affects customers’ attitudes toward community e-commerce platform, we believe that customers’ trust in platforms affects customers’ perceived risk. Figure 1 depicts the overall structure of our research model.

### Effect of e-service quality on customer trust and perceived risk

The effect of the e-service quality of e-commerce platform on customer trust can be explained by the information system model. Applied in the e-commerce context, the information system success model shows that the system quality, information quality, and service quality have an impact on customers’ experience perception (DeLone and McLean, 1992). In particular, in the virtual e-commerce environment, customers may prefer a high level of e-service quality to enhance their transaction confidence in the platform (Luo et al., 2020). For instance, personalized design can stimulate customers’ perceived usefulness and perceived ease of use of e-commerce platform, thus enhancing customer trust (Friedrich et al., 2019). Customers consider such high-quality website services can reduce search costs and transaction costs, and it is one of the trustworthy information sources.

E-service features of different dimensions have a cumulative effect on customer trust (Brengman and Karimov, 2012). For instance, when the system design achieves maximum performance, customers are more likely to get a satisfactory online shopping experience, thereby enhancing customers’ trust in the platform (Hung et al., 2014). Similarly, intelligent fulfillment represents the accurate update and timely transmission of information, which may affect customer trust because it reflects the ability of e-commerce platform e-service to understand customers’ basic needs and desires for consumption (Ahmad et al., 2017). In addition, security assurance is considered as an important factor to protect customers from uncertainty and risks in the consumption process, and customers’ perception of safety and security is crucial to building trust in e-service systems (Shao et al., 2019). Interactive service enables customers to realize timely service contact when encountering problems, so as to correct possible errors in online shopping, and can also bring positive results, such as improved satisfaction (Elsharnouby and Mahrous, 2015). This may increase customers’ willingness to participate in activities and ultimately enhance customers’ trust in the platform. Considering that high e-service quality can affect customer trust, and these effects may accumulate, it can be assumed that a higher level of e-service quality will also be associated with a higher level of customer trust. Therefore, the following hypothesis is proposed:

H1a: System design positively influences customer trust in the platform.

H1b: Intelligent fulfillment positively influences customer trust in the platform.

H1c: Security assurance positively influences customer trust in the platform.

H1d: Interactive service positively influences customer trust in the platform.

In the e-commerce context, Kim et al. (2008) showed that website factors related to security and privacy protection and system quality can reduce the perceived risk of buyers. Factors such as the convenience of interface design affect customers’ perception of the website quality, which in turn affects customers’ perceived risks of its service quality (Hung et al., 2014). The social exchange theory can be used to explain the effect of e-service quality on perceived risk. Generally, individuals can evaluate their expected value extracted from the exchange through comparative cost-reward analysis, which in turn influences their perception and behavior (Priporas et al., 2017). The perception of service quality can be affected by different internal processes, and customers can balance perceived benefits and perceived risks in this process (Mou et al., 2020). Perceived value can be used as an additional theoretical perspective to help understand the potential effect of e-service quality on perceived risk. In the process of interacting with the website, different e-service features can affect customers’ perceived usefulness and perceived enjoyment (Benlian, 2015; Friedrich et al., 2019). These perceptions may negatively affect their perceived risk (Martins et al., 2014).

E-commerce platform reduces perceived risk through various service modes (Mulcahy et al., 2019). For instance, customers can participate in various community activities through the functions of community interaction and group purchases provided by the system design, thereby enhancing their loyalty to the platform. This reduces the customers’ perceived risk to a certain extent (Hasan et al., 2021). Intelligent fulfillment of the community e-commerce platform uses big data technology to improve the authenticity of commodity information and can effectively reduce customers’ perceived risk (Mulcahy et al., 2019). Additionally, the basic service objectives of the e-commerce platform include making customers feel safe and reliable when consuming and helping customers solve problems in time and effectively through interactive service. All these factors ensure that customers can be provided with a pleasant shopping environment, thus reducing customers’ perceived risk (Williams, 2021). It can thus be reasoned that providing high e-service quality will be associated with low perceived risk. Therefore, the following hypothesis is proposed:

H2a: System design negatively influences customer perceived risk.

H2b: Intelligent fulfillment negatively influences customer perceived risk.

H2c: Security assurance negatively influences customer perceived risk.

H2d: Interactive service negatively influences customer perceived risk.

### Effect of customer trust on customer perceived risk

While the connection between perceived usefulness and trust has widely been investigated in the e-commerce literature, different opinions exist on whether customer trust influences perceived risk or perceived risk influences customer trust. In this context, studies focusing on initial online trust argue that when customers may already believe in a shopping platform, customers trust the platform to reduce the risk of technology through security protocols/functions, thereby reducing customer perceived risk (Chen and Chang, 2012; Hansen et al., 2018). Therefore, trust directly affects customers’ attitudes toward the website, and it also affects the overall attitude of the website through perceived risk, presenting a reverse change relationship (Williams, 2021).

The interrelationship between trust and perceived risk exists throughout the customer’s purchase and consumption process (Qalati et al., 2021). At the initial stage of customers’ contact with community e-commerce platform, due to information asymmetry, customers have not formed initial trust in community e-commerce platform, mainly considering whether the purchase and use of products can meet their own needs. Because of the uncertain trust problem, the perceived risk level of customers is rising (Shao et al., 2020). As customers repeatedly contact e-services, if customers find that community e-commerce platform can meet their needs in multiple ways, they will increase trust and thus reduce their perceived risks. In line with studies focusing on initial trust (Brengman and Karimov, 2012; Shao et al., 2019), this study assumes that trust can improve customers’ positive attitude toward the platform, and believe that e-commerce platforms can act for their benefit, which can result in a lower level of perceived risk. Therefore, the following hypothesis is proposed:

H3: Customer trust negatively influences customer perceived risk.

### Effect of customer trust and perceived risk on customer engagement behavior

Initial evidence has also been reported that customer trust in the platform can facilitate the formation of customer engagement. In the e-commerce context, only when customers have enough trust in the e-commerce platform can they engage in social interaction and purchase on the platform for a long time (Hasan et al., 2021; Pop et al., 2021). The trust transfer theory also holds that consumers’ trust in the platform and virtual community can make consumers feel concerned. The formation of this concept positively influences customer engagement behavior (Goraya et al., 2021). Moreover, customers’ trust in the platform can increase website stickiness, which may encourage customers to have an interest in and positive attitude toward the platform, thus being more likely to firmly stick to the platform (Friedrich et al., 2019). Furthermore, Roy et al. (2018a) found a significant positive correlation between trust and customer engagement behavior. Following Roy et al. (2018a), it can be argued that with the increase of customer trust, websites may make customers more comfortable and satisfied, thus stimulating the possibility of customer engagement behavior. Therefore, the following hypothesis is proposed:

H4: Customer trust positively influences customer engagement behavior.

With respect to the effect of perceived risk on customer engagement behavior, Qalati et al. (2021) can show that the higher the perceived risk of consumers using the community e-commerce platform for shopping, the lower the possibility of subsequent purchase and consumption experience. When customers perceive risks beyond their acceptable range, they will reduce their social interaction behaviors or even abandon long-term consumption intentions (Liang et al., 2018). In the e-commerce context, Chiu et al. (2014) found that perceived risk is the result of a comprehensive comparison between the profits and risk losses obtained by customers in the process of purchasing transactions. Once customers understand that using the e-commerce platform may have negative consequences, they avoid it by reducing customer engagement behavior. In addition, Mulcahy et al. (2019) provide evidence that perceived risk can also directly influence customer engagement behavior. In line with Mulcahy et al. (2019), it can be argued that the more customers think that using e-service is risky, the more likely they are to reduce customer engagement behavior. Therefore, the following hypothesis is proposed:

H5: Customer perceived risk negatively influences customer engagement behavior.

### Mediating role of customer trust and customer perceived risk

Community e-commerce divides customers in the form of virtual communities, usually adding personalized interest circles, group buying and other service navigation functions. At the same time, the interaction between customers and members of their community also greatly improves the customer’s perception of service quality (Luo et al., 2020). Customer engagement behavior is mainly generated in the process of interaction between customers and enterprises or other customers. There is preliminary evidence that e-service quality is an important factor affecting customer relationship and can significantly affect customer attitudes and behaviors (Islam et al., 2019). For community e-commerce, customer’s consumption experience has an impact on engagement behavior. Therefore, the e-service quality of community e-commerce platform affects customer behavior, especially customer engagement behavior.

The e-service quality of the community e-commerce platform stimulates customer engagement behavior by improving customer trust. High levels of system design, intelligent fulfillment, security assurance, and interactive service can bring a more convenient and comfortable shopping environment to customers (Elsharnouby and Mahrous, 2015). This effectively reduces customers’ information screening costs in the shopping process and improves decision-making quality (Pop et al., 2021), thereby contributing to a better consumption experience and enhancing customers’ trust in the community e-commerce platform. Customer trust in the platform reduces the uncertainty in the consumption process (Shao et al., 2019; Andronie et al., 2021), prompts customers to actively participate in community activities, and increases the possibility of customer engagement behavior.

The customer’s perceived risk may affect the customer’s expectation of using the community e-commerce platform. By improving the e-service quality of the community e-commerce platform, customers can reduce the perceived risk of the platform and improve the customer perceived value and subsequent willingness to use it (Mulcahy et al., 2019). The system design, intelligent fulfillment, security assurance, and interactive service of the community e-commerce platform enable customers to obtain a reliable e-service experience, reduce risk perception in the process of consumption (Mou et al., 2020), and thus improve customer satisfaction with the community e-commerce platform. On the community e-commerce platform, each dimension of e-service quality has a different influence on customer perceived risk, and if the customer’s risk perception can be reduced, it promotes customer engagement behavior. Therefore, the following hypothesis is proposed:

H6: Customer trust mediates the effects of system design (H6a), intelligent fulfillment (H6b), security assurance (H6c), and interactive service (H6d) on customer engagement behavior.

H7: Customer perceived risk mediates the effects of system design (H7a), intelligent fulfillment (H7b), security assurance (H7c), and interactive service (H7d) on customer engagement behavior.

In conclusion, considering the relationship between e-service quality, customer perception, and customer engagement behavior of community e-commerce platform, based on the above theoretical background and SOR paradigm, this study proposed a research model to investigate how the e-service quality influences customer engagement behavior through customer trust and perceived risk, as shown in Figure 1.

## Research design

### Sample selection

This study adopts the survey method to collect data for empirical tests. This questionnaire includes eight variables, the measurements of which are derived from previous studies. Combining the development characteristics of the community e-commerce platform and the consumer’s consumption experience habits, some items have been fine-tuned to adapt to the context of this study. Since all items were originally developed in English, the forward-backward translation procedure was used to ensure translation accuracy. All the items are translated into Chinese and then back-translated into English. The consistency of the two versions is checked to guarantee that Chinese scales can effectively convey the expected meanings of all the items. In addition, this study made reasonable modifications to the questionnaire through group interviews and guidance from relevant experts to improve the comprehensibility of the questionnaire.

Considering that community e-commerce platforms mainly carry out e-commerce transactions based on virtual communities, this study adopts the form of an online survey. The distribution and collection of questionnaires mainly rely on Wenjuanxing platform^1^, which is among the largest professional research data collection service platform in China. This survey is aimed at customers of community e-commerce platforms, and randomly invited people who have recent online shopping experiences on community e-commerce platforms to distribute questionnaires for the survey. To ensure the quality of the survey sample data, a paid bonus was set up to encourage respondents to fill in the questionnaire carefully. Meanwhile, during the questionnaire collection, the questionnaire whose filling time is less than 90 s is deleted to improve the validity of the survey data. In total, 326 valid questionnaires were received from March to April 2022.

### Variable measurement

The relevant variables in this study adopt a 5-point Likert scale to measure each item (1 = *strongly disagree*, 5 = *strongly agree*), and the respondents are required to choose according to their actual situation. The items of system design, intelligent fulfillment, security assurance, and interactive service are adapted from Wolfinbarger and Gilly (2003) and Luo et al. (2020). The items of customer trust are adapted from Kim and Park (2013) and Möhlmann (2015). The items of customer perceived risk are adapted from Chiu et al. (2014) and Shao et al. (2019). Customer engagement behavior, which includes customer repeat purchase behavior and social interaction behavior, is based on Cambra-Fierro et al. (2013) and Henkens et al. (2021). The control variables in this model include the online experience of using a community e-commerce platform, gender, age, education level, and occupation.

### Sample characteristics

This research uses SPSS23.0 and AMOS21.0 to process and analyze the data, including descriptive statistical analysis, confirmatory factor analysis, structural equation modeling, and mediation effect analysis.

The demographic statistics of the study’s respondents are presented in Table 1. Of all the respondents, 62.2% were female (*n* = 204) and 37.4% were male (*n* = 122). Most of the respondents were ages between 18 and 24 years (*n* = 160, 49.1%), and most have a bachelor’s degree (*n* = 175, 53.7%). The majority of the respondents were enterprise staff (*n* = 147, 45.1%), followed by students (*n* = 88, 27.0%). In terms of customer experience, 53.1% of the respondents have 1–3 years of experience using community e-commerce platforms (*n* = 173, 53.1%).

**TABLE 1:** Demographics of respondents (*N* = *326*).

Measure	Items	Frequency	Percentage
Gender	Male	122	37.4%
	Female	204	62.6%
Age	Under 18	3	0.9%
	18 ∼ 24	160	49.1%
	25 ∼ 30	119	36.5%
	31 ∼ 36	33	10.1%
	37 ∼ 42	5	1.5%
	Over 42	6	1.8%
Education level	High school or below	21	6.4%
	Junior college	68	20.9%
	Bachelor	175	53.7%
	Graduate or above	62	19.0%
Occupation	Student	88	27.0%
	Enterprise staff	147	45.1%
	Government staff	29	8.9%
	Staff of public institution	10	3.1%
	Individuals and freelance	27	8.3%
	Other	25	7.7%
Online experience	6–12 months	88	27.0%
	1–3 years	173	53.1%
	More than 3 years	65	19.9%

## Data analysis

### Measurement model analysis

This research uses Cronbach’s alpha and Composite Reliability (CR) to analyze the reliability of the model. Table 2 shows that Cronbach’s alpha values ranged from 0.804 and 0.890, which is higher than the critical value of 0.7. The composite reliability (CR) values of all variables are between 0.839 and 0.925, which means that the composite reliability of all variables is satisfied. In addition, this research uses average variance extracted (AVE) and item loading to test the validity of the model. The item loadings of all constructs are between 0.713 and 0.887, which exceeds the acceptable level of 0.5, and the average variance extracted (AVE) values are higher than the critical value of 0.5. The results show that the validity of the questionnaire is also satisfied.

**TABLE 2:** Confirmatory factor analysis.

Construct	Items	Mean	*SD*	Standard loadings	Alpha	CR	AVE
SD	SD1	3.71	0.703	0.723	0.881	0.873	0.579
	SD2	3.85	0.793	0.782			
	SD3	3.89	0.796	0.767			
	SD4	3.79	0.879	0.784			
	SD5	4.02	0.854	0.746			
IF	IF1	3.67	0.712	0.731	0.804	0.839	0.637
	IF2	3.97	0.772	0.870			
	IF3	3.95	0.765	0.788			
SA	SA1	3.32	0.939	0.836	0.893	0.874	0.698
	SA2	3.35	0.867	0.819			
	SA3	3.27	0.931	0.852			
IS	IS1	3.67	0.672	0.776	0.821	0.842	0.640
	IS2	3.66	0.756	0.817			
	IS3	3.81	0.742	0.808			
TS	TS1	3.78	0.689	0.878	0.890	0.925	0.753
	TS2	3.65	0.732	0.863			
	TS3	3.74	0.672	0.852			
	TS4	3.78	0.651	0.879			
PR	PR1	2.56	1.082	0.858	0.888	0.923	0.750
	PR2	2.67	0.955	0.870			
	PR3	2.60	1.014	0.887			
	PR4	2.58	0.937	0.849			
CEB	RPB1	3.94	0.697	0.866	0.853	0.905	0.761
	RPB2	3.83	0.765	0.890			
	RPB3	3.93	0.797	0.861			
	SIB1	3.44	0.935	0.714	0.850	0.855	0.541
	SIB2	3.39	0.822	0.716			
	SIB3	3.15	0.934	0.783			
	SIB4	3.28	0.935	0.713			
	SIB5	3.65	0.863	0.751			

All standard loadings are significant at p < 0.001; alpha, Cronbach’s alpha; SD, system design; IF, intelligent fulfillment; SA, security assurance; IS, interactive service; TS, trust; PR, perceived risk; RPB, repeated purchase behavior; SIB, social interaction behavior; CEB, customer engagement behavior.

Furthermore, this research tested the discrimination validity. As shown in Table 3, the square root of each variable’s AVE is higher than the correlation between any two variables. Thus, the discrimination validity of the measurements is confirmed. Moreover, this research also tested the existence of multicollinearity between structures. All the variable inflation factor (VIF) values less than 3.33, which indicates that there is no obvious multicollinearity problem in this study.

**TABLE 3:** Test results of discriminant validity.

Construct	*SD*	IF	SA	IS	TS	PR	RPB	SIB
SD	0.761							
IF	0.488**	0.798						
SA	0.577**	0.428**	0.835					
IS	0.318**	0.487**	0.505**	0.800				
TS	0.454**	0.611**	0.410**	0.633**	0.868			
PR	−0.733**	−0.324**	−0.637**	−0.252**	−0.332**	0.867		
RPB	0.548**	0.488**	0.524**	0.374**	0.497**	−0.510**	0.866	
SIB	0.498**	0.420**	0.582**	0.399**	0.484**	−0.452**	0.540**	0.735

Diagonal numbers are AVE square root, others are correlation coefficients, ** indicates P < 0.01.

Because this study collected data by questionnaire, the validity of the survey data may be affected by common method bias. Therefore, this study collected questionnaires anonymously to ensure the rigor of the research process. In addition, this study also conducted Harman’s single factor test to check for possible method bias. According to the test results, all the extracted factors explained less than 50% of the total variance, and the first variance explained by one factor was 37.751%, indicating that the common method bias of the sample data was not a major concern in this study. At the same time, according to the results of the fit of the one-factor model, χ^2^/df = 2.503 > 3, RMSEA = 0.061 < 0.100, CFI = 0.953, NNFI = 0.924, IFI = 0.934, which are all higher than the minimum fitting standard value of 0.9. This further indicated that there was no common method bias in this study.

### Structural model testing

**FIGURE 2:**
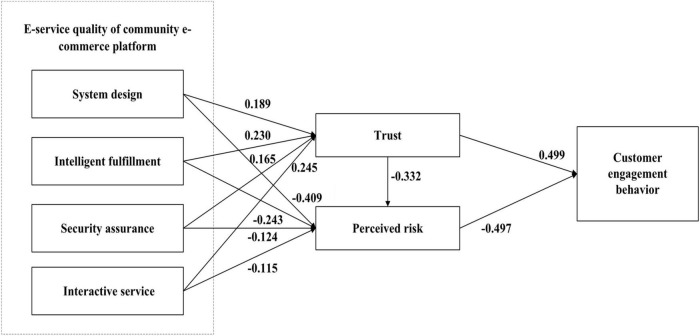
Model testing results.

Figure 2 and Table 4 show the standardized coefficients and significance level of each path in the model. This research first tests the impact of e-service quality on customer trust and the perceived risk of community e-commerce platform. The results show that various dimensions of e-service quality are positively associated with customer trust. System design (β = 0.189, *P* < 0.001), intelligent fulfillment (β = 0.230, *P* < 0.001), security assurance (β = 0.165, *P* < 0.01), and interactive service (β = 0.245, *P* < 0.001) is positively associated with customer trust. These results show that H1a–H1d are all supported. This is in line with similar studies (Kim and Park, 2013; Lin et al., 2017). Consistent with the above hypothesis, various dimensions of e-service quality have a negative impact on customer perceived risk. System design (β = −0.409, *P* < 0.001), intelligent fulfillment (β = −0.243, *P* < 0.001), security assurance (β = −0.124, *P* < 0.01), and interactive service (β = −0.115, *P* < 0.001) is negatively associated with customer perceived risk. These results indicate that H2a–H2d are also supported. Customers are prone to perceived risks due to low e-service quality, in line with similar studies (Martins et al., 2014; Mulcahy et al., 2019; Mou et al., 2020). Therefore, the results show that the e-service quality of community e-commerce platform is positively associated with customer trust, and negatively associated with customer perceived risk.

**TABLE 4:** Structural equation modeling results of the hypotheses.

Hypotheses	Paths	Path coefficients	*T*- values	*P*-values	Supported?
H1a	SD → TS	0.189	3.847	***	Yes
H1b	IF → TS	0.230	5.503	***	Yes
H1c	SA → TS	0.165	3.076	0.003	Yes
H1d	IS → TS	0.245	5.972	***	Yes
H2a	SD → PR	–0.409	–10.074	***	Yes
H2b	IF → PR	–0.243	–5.407	***	Yes
H2c	SA → PR	–0.124	–2.652	0.008	Yes
H2d	IS → PR	–0.115	–2.716	0.006	Yes
H3	TS → CEB	0.499	10.443	***	Yes
H4	PR → CEB	–0.497	10.460	***	Yes
H5	TS → PR	–0.332	–6.342	***	Yes

***P < 0.001.

The results also show that customer trust has a negative impact on customer perceived risk (β = −0.332, *P* < 0.001), thus supporting H3. There is a negative relationship between customer trust and uncertainty perception, which is in line with with previous studies (Mayer et al., 1995; Shao et al., 2019; Vimalkumar et al., 2021). Additionally, the results also provide empirical suggesting that trust is positively correlated with customer engagement behavior (β = 0.499, *P* < 0.001), and perceived risk is negatively correlated with customer engagement behavior (β = −0.497, *P* < 0.001). These findings, respectively, support H4 and H5. Therefore, the results show that customer trust has a positive impact on customer engagement behavior, while perceived risk has a negative impact on customer engagement behavior, in line with similar studies (Chiu et al., 2014; Friedrich et al., 2019; Hasan et al., 2021). Table 4 shows the results of all hypothesis tests. The R-Square of customer engagement behavior is 0.443, indicating that about 44% of customer engagement behavior is explained by customer trust and perceived risk. This empirical result clearly shows that customer perception affects customer engagement behavior in community e-commerce platforms. Similarly, the *R*-Square of trust is 0.536, and the *R*-Square of perceived risk is 0.620, which indicates that system design, intelligent implementation, security guarantee, and interactive service have good explanatory power for customer trust and perceived risk.

### Mediating effect test

This research uses the PROCESS plug-in of SPSS23.0 to test the mediating effects of customer trust and perceived risk based on regression analysis and bootstrapping method. In the present study, the samples of bootstrapping analysis are obtained as 5,000, and the sampling method is the non-parametric percentile method of bias-corrected correction, and the 95% confidence interval is constructed for testing.

The test results are shown in Table 5. The bootstrapping analysis shows that the indirect effect of system design on customer engagement behavior is significantly mediated by customer trust (estimate = 0.135), with a 95% confidence interval excluding zero (CI = 0.122–0.286). The mediating effect of trust between intelligent fulfillment and customer engagement behavior (estimate = 0.221, CI = 0.198–0.453), between security assurance and customer engagement behavior (estimate = 0.118, CI = 0.104–0.252), and between interactive service and customer engagement behavior (estimate = 0.222, CI = 0.223–0.441) are also confirmed. Hence, H6a, H6b, H6c, and H6d are supported.

**TABLE 5:** Test for mediating effects.

	Relations	Direct effect	Indirect effect	Total effect	95% CI	Results
H6a	SD-TS-CEB	0.370***	0.135***	0.505***	[0.122, 0.286]	Supported
H6b	IF-TS-CEB	0.296***	0.221***	0.517***	[0.198, 0.453]	Supported
H6c	SA-TS-CEB	0.391***	0.118***	0.509***	[0.104, 0.252]	Supported
H6d	IS-TS-CEB	0.110***	0.222***	0.332***	[0.223, 0.441]	Supported
H7a	SD-PR-CEB	0.366***	0.140**	0.506***	[0.062, 0.385]	Supported
H7b	IF-PR-CEB	0.386***	0.130***	0.516***	[0.109, 0.289]	Supported
H7c	SA-PR-CEB	0.388***	0.121***	0.509***	[0.076, 0.294]	Supported
H7d	IS-PR-CEB	0.243***	0.089***	0.332***	[0.067, 0.207]	Supported

**P < 0.01,***P < 0.001.

As presented in Table 5, the results show that customer perceived risk plays a partial mediating effect between the four e-service quality dimensions and customer engagement behavior. Therefore, H7a, H7b, H7c, and H7d are supported.

Therefore, findings indicate that customers’ perception of e-service quality of community e-commerce directly and positively affects the main stage of customer engagement behavior, and both customer trust and perceived risk can mediate this process. The results are in line with similar studies, for example (Elsharnouby and Mahrous, 2015; Islam et al., 2019).

## General discussion

Understanding customers’ purchase and participation intention in community e-commerce is particularly important for companies. E-service quality is one of the factors that affect customer engagement behavior. The current research on them mainly focuses on traditional e-commerce. How to provide unique e-service and stimulate customer engagement behavior is of strategic significance to the long-term development of community e-commerce companies. Therefore, this research empirically investigates the impact of the e-service quality of community e-commerce platform on customer engagement behavior.

Based on the SOR model, a mediation model was built in the present study to reveal the impact of e-service quality on customer engagement behavior through customer trust and perceived risk. This study empirically measures the model by surveying customers who have consumed via the community e-commerce platform, and some valuable findings have been drawn. The e-service quality (system design, intelligent fulfillment, security assurance, and interactive service) of community e-commerce platform has a significant impact on customer cognition (including customer trust and perceived risk), which is positively associated with customer engagement behavior. Additionally, the empirical results indicate that customer trust and perceived risk have significant positive impacts on customer engagement behavior, and play a mediating role between e-service quality and customer engagement behavior. More specifically, the e-service quality of community e-commerce platform influences the customer engagement behavior through the partial effect of customer trust and perceived risk.

### Theoretical implications

The results of this research make important contributions to the literature. First, this research employed the SOR model to investigate how e-service quality impacts customer engagement behavior by the mediation effect of customer trust and perceived risk. The present study primarily focuses on the environmental characteristics and individual drivers that stimulate customer engagement behavior in community e-commerce platforms. Previous e-commerce studies have separately examined the important roles of e-service quality and customer perception in shaping the customer decision-making process (Al-dweeri et al., 2017; Qalati et al., 2021). However, the unique e-services of community e-commerce promote customer engagement; thus, customers pay increasing attention to the e-service quality and experience perception. It is necessary to explore the effect of customer perception between e-service quality and customer engagement behavior. Therefore, there are some deficiencies in previous community e-commerce research that this study has somewhat addressed, thus providing a theoretical basis for future studies on customer engagement behavior in community e-commerce.

Second, this research explored the relationship between community e-commerce and e-service quality. With the promotion of precision marketing on the Internet, personalization and communization have changed the way many people live online. Community e-commerce has changed many aspects of traditional e-commerce, but previous studies have not investigated the impacts of these changes (Chen et al., 2015; An et al., 2020). Therefore, the study of customer behavior in community e-commerce platform must consider the stimulating effects of these environmental characteristics. Recognizing the crucial role of e-service quality, this research concentrated on four dimensions, including system design, intelligent fulfillment, security assurance, and interactive service, and validated their effects on customer engagement behavior. This extends prior research on community e-commerce and provides a conceptual foundation for studying the e-service quality in the community e-commerce platform.

Finally, this study recognizes the crucial role of customer trust and perceived risk in influencing the e-service quality of community e-commerce and customer engagement behavior. Because community e-commerce is a form of social commerce with high participation, customer trust and low-risk perceptions are important foundations for customers to participate in community activities and experience consumption. Although some studies have shown that e-commerce platform can build customer trust in social interactions (Kim et al., 2009; Shao et al., 2019), it is still unknown how to build customer trust in the platform and whether customer trust can lead to customer engagement behavior. Additionally, unlike the traditional e-commerce platforms, the e-service characteristics of community e-commerce platform have different impacts on customer trust and perceived risk, thus affecting the stimulation of customer engagement behavior. This study offers a new research perspective for future customer perception of community e-commerce platform.

### Practical implications

The current research presents several practical implications for the management of community e-commerce platforms. First, community e-commerce managers should improve the e-service quality of community e-commerce platform, including system design, intelligent fulfillment, security assurance, and interactive service. For example, they could optimize the system design of community e-commerce platform, and reasonably handle shopping and social functions. Community e-commerce platform companies can make full use of new media such as graphics and text, video, and live broadcasts to enhance customers’ interest, and improve the shopping and social interaction functions such as websites, mobile apps, mini-programs, and video numbers. In addition, they could use high-tech such as artificial intelligence and block chain to strengthen the security and reliability of community e-commerce platform, and they can also subdivide different communities and customers in the community e-commerce platform to improve the response service.

Second, the results of this study show that the e-service quality of community e-commerce platform can positively influence customer engagement behavior through customer trust and perceived risk. Therefore, community e-commerce operators should consider doing so to strengthen customer community belonging. For example, they can regularly investigate experience evaluation through big data analysis and random questionnaires, and timely adjust their marketing strategies. In addition, managers can carry out personalized marketing activities through virtual communities to attract more customers to participate in community interactions. When customers actively participate in social interactions, customers’ trust in the platform can be enhanced, and it is more likely to stimulate customer engagement behavior.

### Limitations and further research

This study has several limitations. First, customer engagement behavior is a dynamic behavior and may evolve over time, as may customer perception and the development of e-service. Thus, future research can consider the longitudinal exploration of customer engagement behavior. Second, this study focuses on the mediating effects of customer trust and perceived risk, and does not further analyze whether this effect is affected by other boundaries, such as corporate social responsibility, corporate reputation and big data management, under the background of digital transformation (Centobelli et al., 2020; Fernando et al., 2022). Regarding these limitations, some future research directions can be identified. For example, how IoT technology can be applied to community e-commerce and e-services can be further analyzed to verify whether and how it affects the long-term effects of customer engagement behavior. Third, this research uses the questionnaire survey method for empirical analysis. Although the measurement items in the survey questionnaire mainly refer to the maturity scale of the previous literature. Thus, it is recommended that future research use experimental methods and the fsQCA method to check the validity of the results and reduce bias.

## Conclusion

With the rapid development of community e-commerce, it has become more and more important to stimulate customer engagement behavior in virtual communities. This research focuses on e-service quality and other factors that promote customer engagement behaviors on community e-commerce platform. Findings indicate that improving the e-service quality (system design, intelligent fulfillment, security assurance, and interactive service) has a strong positive effect on stimulating customer engagement behavior. Furthermore, this study found that customer trust and perceived risk significantly contribute to improvements in customer engagement behavior. The present study advances the current understanding of customers’ activities on community e-commerce platform by empirically showing that e-service quality of community e-commerce platform increases customers’ trust in the platform, and reduces customers’ perceived risk, thereby representing an effective strategy for increasing customer engagement behavior. This study suggests that community e-commerce should improve the e-service quality in system design, intelligent fulfillment, security assurance, and interactive service, cultivate customers’ trust in the platform, and reduce customers’ risk perception of the platform, because these perceptions significantly affect customer engagement behavior.

## Data availability statement

The raw data supporting the conclusions of this article will be made available by the authors, without undue reservation.

## Ethics statement

Ethical review and approval was not required for the study on human participants in accordance with the local legislation and institutional requirements. Written informed consent from the patients/participants or patients/participants legal guardian/next of kin was not required to participate in this study in accordance with the national legislation and the institutional requirements.

## Author contributions

WF contributed to the conceptualization, methodology, statistical analysis, data curation, and writing. BS contributed to the revision, investigation, supervision, funding acquisition, and project administration. XD contributed to the revision. All authors contributed to the manuscript revision and read and approved the submitted version.

## Footnotes

1
https://www.wjx.cn/
